# It is (not) always on Friday: inter-hospital patient transfers in orthopedic and trauma surgery

**DOI:** 10.1007/s00068-023-02335-4

**Published:** 2023-08-20

**Authors:** Jonas Roos, Thomas Loy, Milena M. Ploeger, Leonie Weinhold, Matthias Schmid, Moritz Mewes, Christian Prangenberg, Martin Gathen

**Affiliations:** 1 Department of Orthopedics and Trauma Surgery, University Hospital of Bonn, Venusberg-Campus 1, 53127 Bonn, Germany; 2 Institute for Medical Biometrics, Informatics and Epidemiology, University Hospital of Bonn, Bonn, Germany

**Keywords:** Inter-hospital transfers, Orthopedic complications, Patient flows, Collective analysis, Level I trauma center

## Abstract

**Background:**

While inter-hospital transfers for patients who have suffered major trauma have been well investigated, patient flows for other injured patients, or cases with orthopedic complications, are rarely described. This study aims to analyze the affected collective and to show possible reasons, patterns, and pitfalls to optimize the process in future.

**Materials and methods:**

In a prospective cohort study, all consecutive transfers to a Level I trauma center in Germany were documented and assessed. Patients suffering a major trauma were excluded. Data on the primary treating hospital, patient characteristics, and differences between emergency and elective surgery were analyzed.

**Results:**

A total of 227 patients were included; 162 were injured, while 65 had suffered a complication after elective orthopedic surgery or had a complex orthopedic pathology.

The most common diagnoses leading to transfer were pathologies of the extremities (*n* = 62), pathologies of the spine (*n* = 50), and infections (*n* = 18). The main reasons stated by the transferring hospitals were a lack of expertise (137 cases) and a lack of capacity (43 cases). There was a significantly higher rate of transfers due to trauma (*n* = 162) than for orthopedic patients (*n* = 65), *p* < 0.0001.

**Conclusion:**

There is currently no structured procedure or algorithm for transferring patients in orthopedics and trauma surgery.

## Introduction

Due to changing structures in the health care system, the number of inter-hospital transfers (IHT) has increased [1, 2]. This had led to an increased burden on hospital staff and greater demands on the infrastructure of Level I trauma centers. The most common goal of an IHT is to offer the best center of care for complex or complicated treatments. However, there are known risks involved with discontinuity of care, such as errors in communication, loss of information, and unnecessary repeated examinations [3, 4].

The indication for an IHT and the decision as to which patients should be transferred, and at what time, has not been adequately investigated or standardized for the field of orthopedics and trauma surgery. Very little data are available concerning which patients are selected and why. In the Federal Republic of Germany, no general regulations control patient flow between hospitals. Therefore, the decision as to what clinic patients are sent or recommended can be based on personal, financial, or medical reasons [5, 6].

Furthermore, the IHT itself is often described as a largely unstudied process of care [7]. Adverse events such as inadequate monitoring, equipment failure, and drug errors can occur more commonly during an IHT than during in-hospital treatment [8, 9]. In contrast to transfers of complicated orthopedic cases or minor traumatic injuries, there are well-established trauma networks which aim to provide a high-level quality of medical care after a major trauma, despite regional differences in infrastructure, technical equipment, or human resources [10]. Studies have shown beneficial treatment and significant improvement in the probability of surviving a major trauma after the establishment of trauma networks [11–14].

Overcrowded emergency units that provide delayed care to patients can be symptomatic of an overburdened and organizationally suboptimal healthcare system. It is important to prevent inappropriate transfers so as to minimize the increased strain on resources at Level I trauma centers [5, 9]. This study was set up to understand the prevalence of IHT and to improve the transfer process. The aim is to assess characteristics of transferred patients, reasons for the hospital not being able to treat the patient, and to determine the health outcomes of the patients.

## Materials and methods

### Study design

The researchers conducted a prospective cohort study of inter-hospital patient transfers in orthopedic and trauma surgery from April 2021 to January 2022. The investigated hospital is a 1200-bed university clinic and the only Level I trauma center in a metropolitan area with a population of almost 1 million people. The hospital is part of a trauma network that includes nine hospitals, four Level II trauma centers, and four Level III trauma centers. The clinic has further expertise in arthroplasty and spine surgery, is a center for geriatric traumatology, and is a certified cooperation partner of the German Cancer Society. Patient data were evaluated from the electronic patient files of the hospital as well as from records supplied by the transferring hospitals. The study was approved by the local institutional review board (No. 157/21).

### Data source

Patient data were accessed via ORBIS® (Agfa HealthCare, Mortsel, B), an electronic data management system. We extracted and reviewed the collected data from our hospital, as well as data from the external referring physicians.

### Inclusion and exclusion criteria

All patients transferred to our clinic from April 2021 to January 2022 were included in the study. The study comprised patients who were transferred on the day of the request, as well as those scheduled to be admitted on the next working day. The classification of whether the transfers took place during regular working hours or during on-call hours was determined based on the arrival time of the patients at the clinic. Transfer requests that could not be finalized were not included in this study. All requests were accepted for patients who did not require further intensive medical monitoring. In cases of temporarily overwhelmed intensive care capacity, individual patient transfers were denied. Patients who did not require inpatient admission after examination and treatment were also excluded from the study. Likewise, all patients who had suffered a polytrauma (ISS ≥ 16) were excluded.

### Data extraction

Initially, patients' demographic data were extracted from ORBIS®, and included age, gender, American Society of Anesthesiologists Classification (ASA score), Injury Severity Score (ISS score), and insurance status. In addition, data on hemoglobin (Hb) and c-reactive protein (CRP) were recorded on admission and at discharge; also recorded were number of surgeries, location of injuries, number of pre-existing conditions, number of medications, isolation status of patients, and whether documentation was complete at the time of transfer. In addition, we recorded pre-hospital data: which hospital the patient was transferred from, the time point and medical reasons for transfer, and whether there were existing medical complications. Also recorded were length of stay, DRG amount, and subsequent care.

Furthermore, like Goldfarb et al., who attempted to evaluate the appropriateness of transfers, we also investigated which transfers to a maximum care provider were justified. Goldfarb et al. developed a score ranging from 0 to 10, analogous to a visual analog scale, in which transfers with a score of 0 were completely inappropriate, while those with a score of 10 were completely justified. A score of 1, for example, was assigned to a simple closed fracture that did not require emergency treatment, while a score of 10 represented complex orthopedic procedures that required immediate treatment [6].

A similar study was conducted by Crichlow et al., who also examined the appropriateness of transfers for orthopedic injuries and additionally analyzed the physician's specialization as a possible risk factor for transfers [15]. It is noteworthy that in both studies, a large proportion of transfers were carried out by non-orthopedic physicians, leading to a significant lack of expertise and limited treatment options. In this study, the assessment of appropriateness was carried out retrospectively by the authors using the score by Goldfarb et al. Trauma surgery transfers with a VAS score of less than 5 according to Golfarb et al. and an ASA score of less than 3 were deemed inappropriate. Here, one of the two criteria was sufficient. The same criteria were applied to orthopedic transfers, with a focus on an ASA score of less than 3 and the treatment diagnosis. Since the VAS score according to Goldfarb et al. primarily considers the injury pattern, it was not applied to orthopedic transfers. Only transfers with low treatment severity were included as inappropriate in this context.

The times of the transfers were divided into regular work from Monday to Friday from 8 a.m. to 4 p.m. and time on standby, which was Monday through Thursday from 4 p.m. to 8 a.m. and Friday from 4 p.m. to Monday at 8 a.m.

### Statistical analysis

Characteristics of the data are described using means with standard deviations (SD) or medians with interquartile range (IQR) for continuous variables and frequency distributions with percentages for categorical variables. The outcome variable was defined as the number of patient transfers to the department of orthopedics and trauma surgery at our hospital. Differences between orthopedics and trauma surgery regarding patients’ characteristics or patient transfers were assessed by Chi-squared tests (categorical variables) and the Wilcoxon Mann–Whitney *U* test (continuous variables). The Bonferroni method was used to correct *P*-values for multiple testing.

The differences between the number of trauma surgery and orthopedics patient transfers, and whether there were similar numbers of patient transfers within and outside of core working hours, were assessed by the binomial test.

*P*-values < 0.05 were considered significant. All analyses were carried out using the R Software for Statistical Computing Version 4.1.2.

## Results

### Background characteristics

Comparisons of baseline characteristics between orthopedics and trauma surgery patients are summarized in Table 1.

**Table 1:** Demographic data of the patients broken down by orthopedic and trauma surgery transfers

	Orthopedics	Trauma surgery	Total	*p*-value
Age (years)				
Mean (SD)	72.7 (11.6)	62.7 (22.6)	65.5 (20.5)	0.021
CRP (mg/L) (admission)				
Median (IQR)	53.1 (15.4–139.4)	27.8 (6.0–68.6)	37.4 (6.5–88.9)	0.106
CRP (mg/L) (discharge)				
Median (IQR)	29.2 (10.4–47.5)	27.0 (9.4–49.0)	27.0 (9.5–48.1)	1
Hemoglobin (g/dL) (admission)				
Median (IQR)	11.3 (9.9–12.3)	12.3 (10.4–14.0)	12.0 (10.3–13.4)	0.084
Hemoglobin (g/dL) (discharge)				
Median (IQR)	10.1 (9.2–11.2)	11.0 (9.5–12.6)	10.8 (9.4–12.0)	0.499
ISS				
Median (IQR)		9.0 (9.0–9.0)		
Drugs (*n*)				
Mean (SD)	8.0 (4.8–13.3)	4.0 (0.0–9.0)	5.0 (1.0–10.0)	< 0.001
Pre-existing conditions (*n*)				
Mean (SD)	5.0 (3.0–8.0)	2.0 (1.0 –5.0)	3.0 (1.0–6.0)	< 0.001
Isolation *n* (%)				
No	60 (92.3)	155 (95.7)	215 (94.7)	1
Yes	3 (4.6)	6 (3.7)	9 (4.0)	
(Missing)	2 (3.1)	1 (0.6)	3 (1.3)	
Insurance status *n* (%)				
Statutory health insured	50 (76.9)	122 (75.3)	172 (75.8)	1
Privately insured	10 (15.4)	17 (10.5)	27 (11.9)	
Statutory accident insurance	1 (1.5)	18 (11.1)	19 (8.4)	
Self-payer	2 (3.1)	2 (1.2)	4 (1.8)	
(Missing)	2 (3.1)	3 (1.9)	5 (2.2)	
Surgeries (external, [*n*])				
Mean (SD)	0.3 (1.1)	0.1 (0.5)	0.2 (0.7)	1
Surgeries (internal, [*n*])				
Mean (SD)	1.4 (1.5)	1.1 (1.2)	1.2 (1.3)	1

A total of 227 patients were included in the study; 162 patients (71.4%) were transferred because of a traumatological etiology and 65 suffered a complication after elective orthopedic surgery or had a complex orthopedic pathology. There was a significant group difference in age between the orthopedic (72.72 ± 11.6 years) and trauma surgery (62.7 ± 22.6 years) transfers, with the orthopedic patients being significantly older (*p* = 0.021). There were no significant differences in patients’ CRP level (admission *p* = 0.106; discharge *p* = 1) and hemoglobin level (admission *p* = 0.084; discharge *p* = 0.499) at admission and discharge. Overall, the data show decreasing infection levels between admission and discharge, with constant hemoglobin levels. The median ISS for the trauma patients was 9 points (IQR: 4.0–9.0). There was a significant difference between the orthopedic and trauma surgical transfers in terms of medications taken (*p* < 0.001) and pre-existing conditions (*p* < 0.001). The orthopedic patients took more medications (mean 5.0 vs. 2.0 in trauma transfers) and had more pre-existing conditions (mean 5.0 vs. 2.0 in trauma transfers). There was no significant difference between the number of isolated patients in orthopedics and trauma surgery. Overall, 3.6% of patients had to be isolated. With a multidrug-resistant population of up to 9.5%, this percentage is relatively low [16]. Information for three patients was missing. There was no difference between orthopedic and trauma surgical patients in terms of external and internal operations and no significant difference between the groups in insurance status (*p* = 1). Among the transfers, 45 patients (19.8%) received conservative therapy, while 58 patients (25.5%) underwent emergency surgery on the same day. The remaining 124 patients underwent surgery after a median of 2 days. The distribution of patients for the ASA-Score is shown in Table 2. Most patients had an ASA score of 3 (41.4%), followed by an ASA score of 2 (35.6%). An ASA score of 1 was the least frequent (7.9%). The ASA score could not be obtained for four patients.

**Table 2:** Distribution of ASA scores for the orthopedic and trauma surgery transfers

ASA (admission) *n* (%)	Orthopedics	Trauma surgery	Total
1	2 (3.1)	16 (9.9)	18 (7.9)
2	8 (12.3)	73 (45.1)	81 (35.7)
3	43 (66.2)	51 (31.5)	94 (41.4)
4	9 (13.8)	20 (12.3)	29 (12.8)
5	1 (1.5)	0 (0.0)	1 (0.4)
(Missing)	2 (3.1)	2 (1.2)	4 (1.8)

The corresponding percentages are given in parentheses

**Fig. 1:**
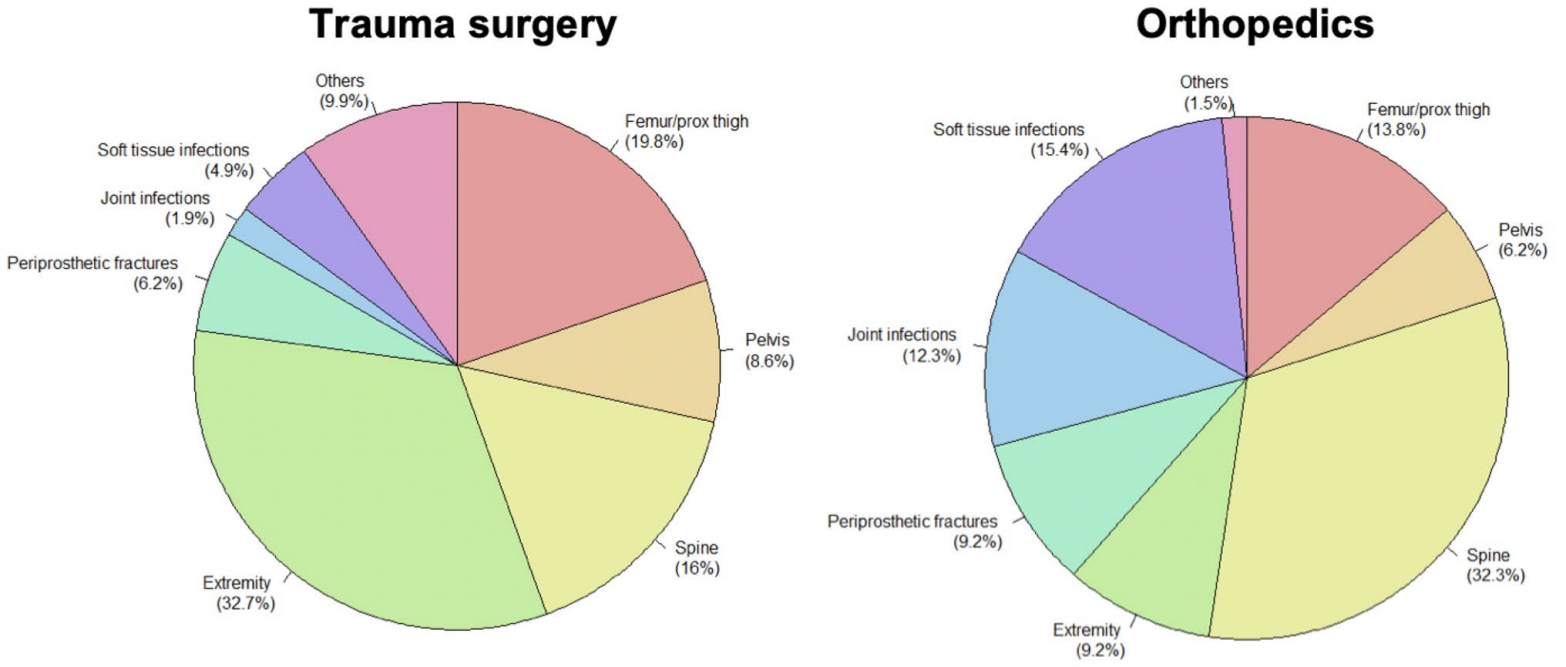
The percentage distribution of the most common injury patterns in trauma surgery and orthopedics. The allocation concerning periprosthetic fractures and joint infections to orthopedics or trauma surgery depends on the original indication for joint replacement (fracture vs. osteoarthritis) according to in-house regulation. Fractures of the spine were divided into result of an adequate trauma (trauma surgery) vs. result of an inadequate trauma (orthopedics). Soft tissue infections were allocated according to in-house rules (e.g., soft tissue infections of the hand belong to trauma surgery)

The most common diagnoses of transferred patients are shown in Fig. 1. The main cause of trauma surgery transfers was injuries to the extremities, including a large proportion of hand injuries. Orthopedic transfers primarily required further treatment for spinal conditions. At 38%, spondylodiscitis was the most common cause of spinal disorders.

### Transfers

**Fig. 2:**
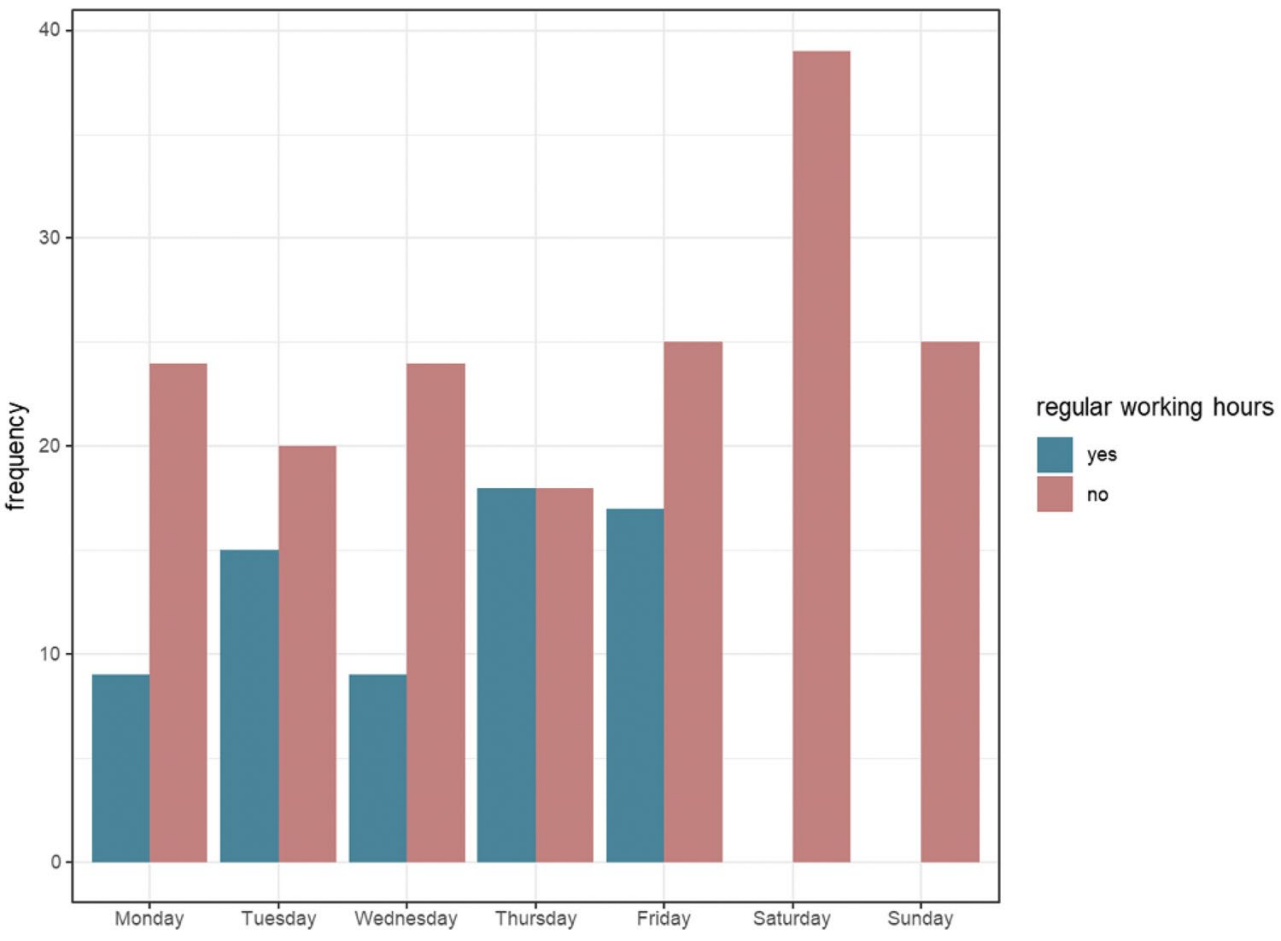
Distribution of all transfers in orthopedics and trauma surgery combined, stratified by working hours and days of the week. Blue represents core working hours and red on-call duty hours

The most common diagnoses that led to a transfer were pathologies of the extremities (*n* = 55), pathologies of the spine (*n* = 46), and infections (*n* = 18). In this context, the spondylodiscitis cases were classified as spinal disorders. The main reasons stated by the transferring hospitals were a lack of expertise in 123 cases (55.2%) and a lack of capacity in 40 cases (17.6%). Lack of capacity includes lack of intensive care beds, lack of anesthesiologic experience, and lack of capacity in the operating theater. There was a significantly higher rate of transfers due to a trauma (*n* = 162, *p* < 0.001). Figure 2 shows the number of transfers during working hours and on-call duty distributed over the days of the week.

**Fig. 3:**
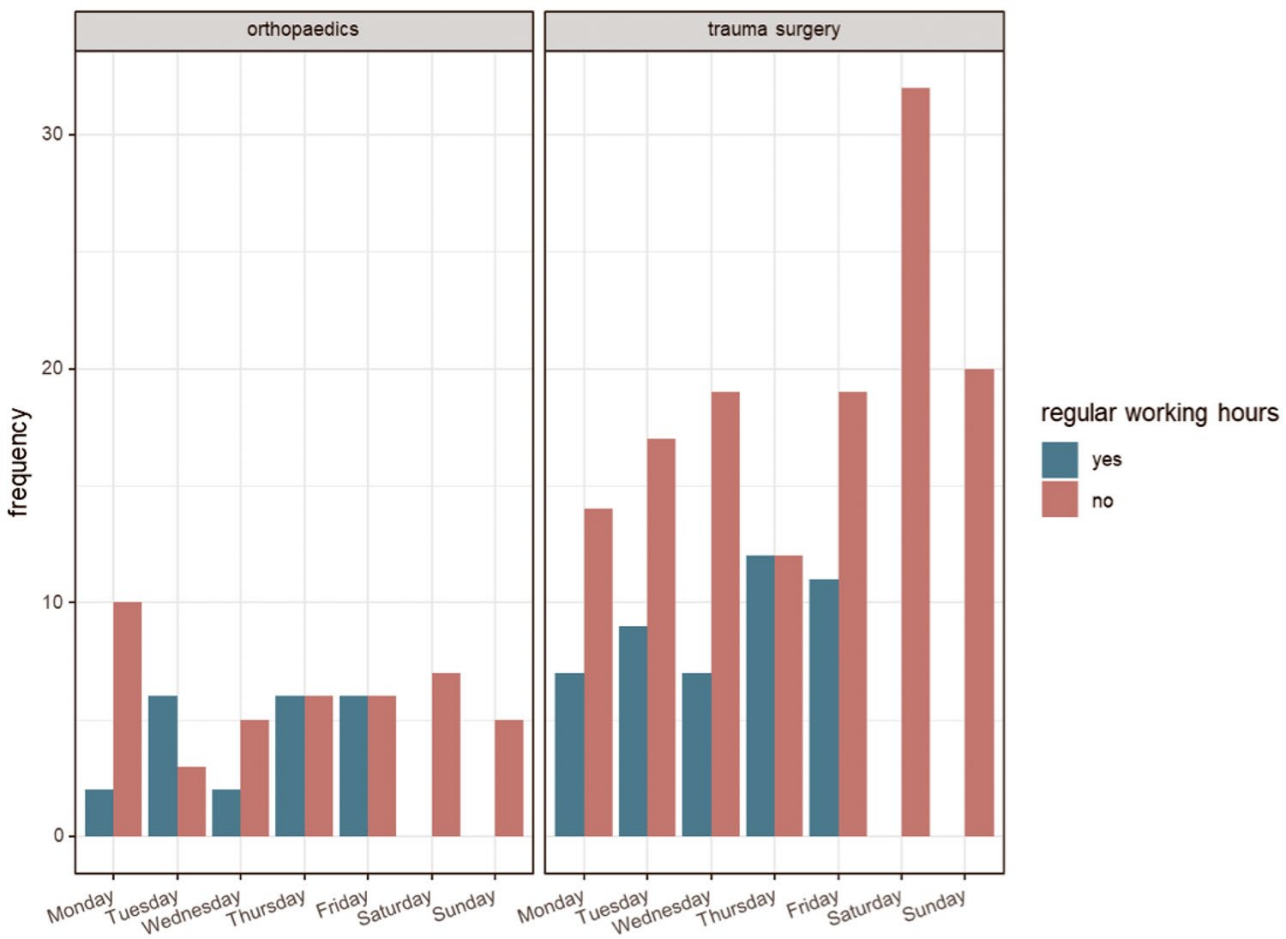
Distribution of transfers in orthopedics and trauma surgery, stratified by working hours and days of the week. Blue represents core working hours and red on-call duty hours

Of all patient transfers, 71% were transferred during on-call time and 29% during work time. This revealed a significant difference (*p* < 0.001): a predominant proportion of transfers were conducted outside regular working hours. Likewise, a significant difference (*p* < 0.0001) was found between transfer times in orthopedics and in trauma surgery (see Fig. 3).

A large proportion of patients came from small local centers (*n* = 126) and from regional centers (*n* = 69). Most of these were assigned to the responsible trauma network and were within the urban area. Of the 227 patients transferred, 49 had incomplete diagnostics (21.6%), with the proportion in orthopedics slightly lower (22.6%) than in trauma surgery (29.6%). The largest proportion of patients could be transferred to the regular ward. Only 5.7% required immediate intensive medical monitoring**.**

### Appropriateness of transfer

Nearly all of the transfers in our study came from orthopedic and trauma surgery clinics or were patients who had already been evaluated in the emergency department by physicians in these fields. Therefore, appropriateness in our study was primarily determined in reference to the Goldfarb score, based on the pattern of injury and the patient’s ASA score.

The proportion of inappropriate transfers was 18.5% (42 of 227). Of the orthopedic transfers, 7% were inappropriate, while 22.7% of the trauma transfers were inappropriate. The largest percentage of these trauma surgical transfers involved hand injuries or minor non-septic soft tissue infections of the upper extremities (24.4%). Of these patients, 35% were managed conservatively, while 28% underwent surgery on the same day. The remaining patients underwent surgical intervention, with a median time to surgery of 1.5 days. The patients had an ASA score of 1 or 2, and none had suffered a severe injury, such as an amputation (Table 3).

**Table 3:** Comparison of the key characteristics of the patients allocated to inappropriate transfer (broken down by orthopedic and trauma surgery transfers) vs. appropriate transfer concerning metrics for Goldfarb score

	Inappropriate orthopedic	Inappropriate trauma surgery	Appropriate
Age (years)			
Mean (SD)	69.2 (22.9)	46 (21.1)	69.4 (18.5)
VAS score according to Goldfarb et al.			
Median (IQR)		2 (0–4)	6 (4–8)
ASA score			
Median (IQR)	2 (1–3)	2 (1–3)	3 (2–4)

VAS was stated for trauma patients following the study of Goldfarb et al.

### Treatment of patients

The median length of stay was 10 days, with no significant difference between orthopedic and trauma surgery patients (*p* = 0.108). Of the transferred patients, 5.7% had already suffered a complication externally. During their stay, 16% of the patients experienced a complication. The most frequent complications were nosocomial pneumonia and urinary tract infections. On average, the patients underwent one operation in our hospital (median).

The median DRG reimbursement was 8188€. DRG reimbursement for orthopedic transfers was significantly higher (13014€) compared to trauma surgery (7129€) (*p* = 0.027).

Following inpatient treatment, most patients were discharged home (*n* = 88). A large proportion of patients were transferred to inpatient geriatric treatment (*n* = 84). Ten patients (4.4%) died during their stay.

## Discussion

Our data showed significantly more transfers due to a trauma than after elective orthopedic surgery (trauma: *n* = 162, orthopedic: *n* = 65, *p* < 0.0001) Lack of expertise was given as the most common reason for a transfer in 54.2% of all cases, followed by a lack of capacity to properly treat the patient (17.4%). It is notable that lack of experience was more often significant in elective orthopedic cases (70.7%) compared to trauma cases (47.5%).

Another notable finding was the low number of surgeries in external hospitals (*m* = 0.2, SD = 0.7) and the high number of patients promptly transferred. The question arises as to whether primary admission to the hospital made sense initially so as to prevent unnecessary transfers. Pre-hospital transfer strategies are a controversial topic that has been investigated in multiple studies, especially concerning patients suffering a major trauma. Some authors found a reduction in morbidity and mortality when patients who had suffered major trauma were transported directly to a Level I trauma center, while other studies found no significant difference concerning the outcome after initial treatment in a local hospital [17–19]. In comparison to trauma cases, there are few recommendations and little research on transfer strategies in complicated orthopedic cases, such as a periprosthetic joint infection or spondylodiscitis. Although treatment in a specialized center is often recommended, our results show that the patients were at different stages of diagnosis and therapy when they were admitted.

Allocation and triage must be precisely organized to guarantee the best possible treatment and to prevent trauma centers from being overwhelmed by the assessment of minor injuries that could reasonably be treated in a primary care hospital [20]. The question of whether a transfer is appropriate or not depends on multiple factors and is not easy to answer. Some studies have concluded that a secondary overtriage is an unnecessary transfer of patients to another hospital. Overtriage refers to patients with an ISS lower than 16, who do not need an operative intervention, and who can be discharged within 48 h after admission [21, 22]. A study analyzing 7793 patients found a secondary overtriage in 24% of adult and 49% of transferred pediatric patients. The authors concluded that additional costs and resource utilizations are a relevant effect of the findings and that local organization structures need to be established to reduce overtriage [23].

**Fig. 4:**
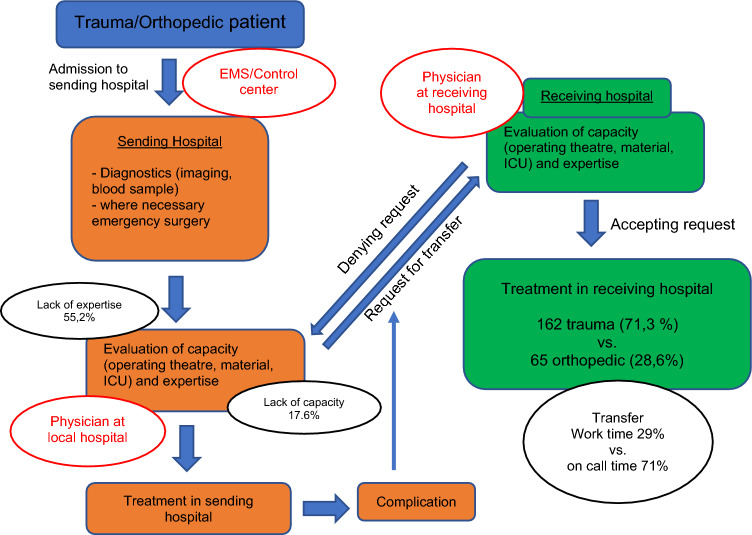
Process of transfers of trauma and orthopedic patients. Red text boxes indicate decision makers, either preclinical or in the treating hospitals. Most of the decisions made during inter-hospital transfers are rather freestyle and not standardized

The process of transferring patients in trauma surgery and orthopedics for non-polytraumatized patients, for example due to lack of capacity or expertise, currently does not follow an ordered process. There are currently neither recommendations on which patients should be transferred nor a concept of how these transfers can be regulated. Figure 4 details the current transfer process from the admission of a trauma or orthopedic patient to the sending hospital up until the treatment of the transferred patient in the receiving hospital. The first decision is made by the emergency medical service and control center, which allocates patients to certain hospitals. Depending on the injury pattern, the control center decides which specialties and expertise are needed for appropriate treatment of the patient. This decision is made on a rather ad hoc basis, and could lead to overtriage or undertriage. In addition, capacity in intensive care unit, operating theater, and expertise are often not considered. After admission, diagnostics, and, if needed, emergency surgery, the physician at the sending hospital must determine if capacity and expertise are sufficient to treat the patient adequately. This determination is not standardized. If there is a lack of capacity or expertise, the physician at the sending institution must request multiple hospitals for transfer. This takes time and leads to delays in the well-timed treatment of the patient. To improve the process of transferring patients, standardized and automatized steps are needed, such as a digital platform for requesting multiple hospitals for transfer at the same time. Also useful would be a digital application that could indicate each hospital’s surgery expertise, as well as live updates concerning capacity in the operating theater. Such a tool already exists for displaying capacity in the intensive care unit.

Using a visual analog scale introduced by Goldfarb et al. [6], we defined 42 cases (18.5%) as not appropriate for transfer. The analog scale utilizes data from common patient transfers as a means of comparing case complexity and the need for referral. The included patients showed mostly low ASA scores and had minor injuries or septic soft tissue infections without systemic infection that did not need treatment in a Level 1 trauma center. Thakur et al. investigated 260 transfers to a Level I center and concluded that 52% were inappropriate [5]. The definition of an inappropriate transfer was made solely by analyzing the diagnosis and deciding if it could be managed by a certified trained general orthopedist in the community. However, there was no insight into the resources, equipment, and staffing of the peripheral hospital. As a result, the transfer was deemed unsuitable based on a one-sided and subjective evaluation. Furthermore, the authors found high rates of uninsured patients when the transfer was due to a benign orthopedic injury or disorder, suggesting that financial interests may have played a role in the selection. In our cohort, the rate of privately insured patients was 11.2% and the rate of mandatory health insured patients was 74.4%. Our findings do not suggest that financial motives were a factor in the transfers, as the values observed were within the range of those seen in the general population. A similar study conducted by Chrislow et al. [15] analyzed the prospective data of 546 patients transferred to a Level I trauma center; 16.5% of the transfers were deemed completely inappropriate, 34.2% were designated as intermediate, and 49.3% as appropriate. A visual analog scale ranging from 0 to 10 was used to evaluate the appropriateness of patient transfers [6]. The main reason for the transfers was attributed to a lack of expertise. This matches with our findings, in which 55.5% of transfers were due to a lack of experience. Watson analyzed and compared the studies of Thakur and Chrislow and found that both studies stated that transfers were more likely to happen on weekends than on normal working days. Both studies further showed a trend toward transfers occurring after routine working hours [24]. The effect was also confirmed by our data, in which 71% (*p* = 0.0001) of transfers occurred outside of core working hours and had to be handled by the on-call surgical team. The resulting intensification of work during calls may lead to job dissatisfaction or even scarce resources in the affected hospitals [25, 26]. Watson’s view echoes the sentiment of numerous orthopedic surgeons practicing at Level I trauma centers. Many local or community hospitals do not wish to manage even the most basic aspects of orthopedic trauma. As a way to improve the process, he suggests a mandatory and respectful discussion with the referring institution’s in-call orthopedist. Further studies also mentioned effective communication between referring and receiving medical staff as imperative to ensuring a safe IHT [9, 27]. This includes specific transfer letters and tools such as pretransfer checklists or teleradiology [28, 29].

This study has several limitations. First, it is a one-center study involving a relatively low number of cases compared to the overall numbers of IHTs. Therefore, it includes no horizontal transfers, but only vertical transfers from Level II or III centers to a Level I trauma center. Due to the heterogeneous cohort and missing follow-up examinations, it is difficult to make generally valid statements about outcomes after IHT.

In conclusion, inter-hospital transfers are neither registered nor regulated across the board. Therefore, a targeted distribution according to specialization and resources is not made. This leads to a high number of costly transfers and unnecessary short stays in peripheral hospitals. A concept is needed that systematically distributes not only polytrauma patients but also patients with other complex orthopedic disorders.

## Data Availability

Data available on request due to privacy/ethical restrictions.
